# Editorial: Mechanical and genetic signaling in striated muscle development, aging and disease

**DOI:** 10.3389/fphys.2024.1376066

**Published:** 2024-02-09

**Authors:** Claudia Crocini, Emma L. Robinson, Joseph D. Powers

**Affiliations:** ^1^ Max Rubner Center (MRC) for Cardiovascular, Metabolic and Renal Research, Charité -Universitätsmedizin Berlin, Berlin, Germany; ^2^ German Center for Cardiovascular Research (DZHK), Berlin, Germany; ^3^ Division of Cardiology, Department of Medicine, University of Colorado Anschutz Medical Campus, Aurora, CO, United States; ^4^ Department of Mechanical Engineering, University of Washington, Seattle, WA, United States; ^5^ Department of Laboratory Medicine and Pathology, University of Washington, Seattle, WA, United States

**Keywords:** striated muscle, development, disease, genetics, epigenetics

Human striated muscles are made of multiple cell types, forming highly structured and dynamic tissues that provide specific critical tasks in our daily lives—from pumping blood throughout our bodies with each heartbeat to enabling movement and communication. As striated muscles contract, sarcomeric forces produced in the myocytes necessarily pass throughout and between cells, as well as scale up from the single-cell level to the whole organ level. Passive mechanical properties of both the intracellular and extracellular environments also contribute to the myriad of mechanical signals that are constantly being transmitted throughout striated muscles. Moreover, mechanical signals that reach the cell nucleus can also cause changes in the nucleus at the level of the epigenome, affecting chromatin organization and accessibility, mediating a mechano-sensitive regulation of gene expression (Alisafaei et al., 2023). Thus, the proper regulation of mechanical signals in muscles is likely fundamental to healthy muscle structure and function.

It is well established that changes at the level of chromatin is altered in the context of cardiovascular and skeletomuscular disorders including heart failure and muscular dystrophies (Kim et al., 2016; Rugowska et al., 2021). Understanding the role of mechanosignaling pathways in regulating healthy muscle function and development, and to what extent abnormalities in these pathways leads to age- and disease-related muscle dysfunction, is currently a major focus of biomedical research. Recent technological advances in biomedical imaging modalities, next-generation sequencing, and computational modeling have enabled unprecedented insights into the intricate world of mechanical and genetic signaling within striated muscles. The goal of this Research Topic was to collect current research and ideas that advance our understanding of mechanical, genetic, and epigenetic signaling in cardiac and skeletal muscle development, aging, and disease, as well as the *in vitro*, *in vivo*, and *in silico* models that can be used to study these mechanisms.

In one study published in this Research Topic, Mazzaro et al. developed new methodology for analyzing murine and human muscle sympathetic innervation. The authors compared the structure of the sympathetic neuronal network in healthy muscles and muscles with amyotrophic lateral sclerosis (ALS), a deadly neuromuscular disease. Through their new quantitative imaging approach, they show that sympathetic neurons are compromised in a mouse model of ALS, and that the sympathetic neuron denervation occurs early in disease progression.

In another study, Pan et al. investigated the role of RNA networks in skeletal muscle development by performing RNA sequencing with leg muscles from embryonic chickens. The authors identified thousands of differentially expressed RNA transcripts between muscles from E10 and E18 chickens, including mRNA, miRNA, long non-coding RNA (lncRNA), and circular RNA (circRNA). They used this information to construct a predictive regulatory network of competing endogenous RNA interactions in developing muscle, which can shed light on key RNA interactions that regulate gene expression during healthy skeletal muscle development.

One study investigated the impact of a hypertrophic cardiomyopathy (HCM) mutation on cardiac troponin T (cTnT-R92Q) at early postnatal days with the goal to identify mechanisms involved in the early progression of the disease Langa et al. Already at 7 ays after birth, mice showed diastolic dysfunction with altered coronary flow, likely due to changes in endothelial YAP signaling, and increased fibrosis. This work emphasizes the important crosstalk between cardiac myocytes carrying the HCM mutation and other cellular populations and compartments of the heart the disease progression.

Since mutations in ribosomal protein L3-like (RPL3L) are associated with childhood cardiomyopathy, Grimes et al. investigated the effects of RPL3L deletion in mouse hearts. The authors identified a compensatory mechanism by the paralogue RPL3 but a role for RP3L in reducing cardiac growth with aging. This work suggests that mutations in RPL3L associated with childhood cardiomyopathy may not act as loss of function but may involve alternative mechanisms.

Additionally, this Research Topic encompasses various reviews addressing mechanosignaling in cardiomyopathy and skeletal myopathy, as well as the epigenetic relationships with metabolism in muscle and mechanical performance of peripheral muscles after COVID-19 infection. These works collectively synthesize recent research, providing valuable perspectives and contributing to the evolving understanding of muscle biology.
